# Open-Gated pH Sensor Fabricated on an Undoped-AlGaN/GaN HEMT Structure

**DOI:** 10.3390/s110303067

**Published:** 2011-03-09

**Authors:** Mastura Shafinaz Zainal Abidin, Abdul Manaf Hashim, Maneea Eizadi Sharifabad, Shaharin Fadzli Abd Rahman, Taizoh Sadoh

**Affiliations:** 1 Material Innovations and Nanoelectronics Research Group, Faculty of Electrical Engineering, Universiti Teknologi Malaysia, 81310 Skudai, Johor, Malaysia; E-Mails: mastura@fke.utm.my (M.S.Z.A.); maneea.eizadi@gmail.com (M.E.S.); shaharinfadzli@fke.utm.my (S.F.A.R.); 2 Department of Electronics, Kyushu University, 744 Motooka, Fukuoka 819-0395, Japan; E-Mail: sadoh@ed.kyushu-u.ac.jp (T.S.)

**Keywords:** AlGaN/GaN, pH sensor, open-gated structure, liquid-pHase, HEMT

## Abstract

The sensing responses in aqueous solution of an open-gated pH sensor fabricated on an AlGaN/GaN high-electron-mobility-transistor (HEMT) structure are investigated. Under air-exposed ambient conditions, the open-gated undoped AlGaN/GaN HEMT only shows the presence of a linear current region. This seems to show that very low Fermi level pinning by surface states exists in the undoped AlGaN/GaN sample. In aqueous solution, typical current-voltage (*I-V*) characteristics with reasonably good gate controllability are observed, showing that the potential of the AlGaN surface at the open-gated area is effectively controlled via aqueous solution by the Ag/AgCl gate electrode. The open-gated undoped AlGaN/GaN HEMT structure is capable of distinguishing pH level in aqueous electrolytes and exhibits linear sensitivity, where high sensitivity of 1.9 mA/pH or 3.88 mA/mm/pH at drain-source voltage, *V_DS_* = 5 V is obtained. Due to the large leakage current where it increases with the negative gate voltage, Nernstian like sensitivity cannot be determined as commonly reported in the literature. This large leakage current may be caused by the technical factors rather than any characteristics of the devices. Surprisingly, although there are some imperfections in the device preparation and measurement, the fabricated devices work very well in distinguishing the pH levels. Suppression of current leakage by improving the device preparation is likely needed to improve the device performance. The fabricated device is expected to be suitable for pH sensing applications.

## 1. Introduction

Many semiconductor materials have been tested for their suitability as ion sensors; in particular there is an emerging interest in the use of wide band gap semiconductors as sensitive chemical sensors. Gallium nitrides (GaN) are chemically stable semiconductors with high internal spontaneous and piezoelectric polarization, which make them very suitable materials to create very sensitive but robust sensors for the detection of ions, gases and polar liquids, particularly at high temperatures and in harsh environments [1,2]. AlGaN/GaN high-electron-mobility-transistor (HEMT) structures have been extremely useful as gas and liquid-phase sensors due primarily to three reasons: (1) a high electron sheet carrier concentration channel induced by piezoelectric polarization of the strained AlGaN layer [3], (2) the carrier concentration which is strongly depends on the ambient conditions [2,4] and (3) an opportunity for on-chip co-integration with signal processing and communication circuits [5]. In addition, sensors fabricated from these wide band-gap semiconductors could be readily integrated with solar blind UV detectors or high temperature, high power electronics with wireless communication circuits on the same chip to provide high speed transmission of the data.

Work on the pH responses to n-doped AlGaN surfaces was recently reported by Kokawa *et al*. [6] utilizing an open-gated structure. The possibility of pH sensing using undoped AlGaN/GaN HEMT has been reported previously by Mehandru *et al.* [7]. The fabricated two-terminal device shows significant decreases upon exposure of the gate area to solvents (water, acetone and HCl) [7]. Recently, we have also reported the sensing responses of both bulk n-GaN and undoped AlGaN/GaN based two-terminal devices in aqueous solution with various pH levels and also in polar liquids [8]. There are some major works that study the effect of oxide layers on open-gated AlGaN/GaN HEMT devices [9,10]. However, in reality, the mechanism of the pH response to such GaN and AlGaN surfaces is not well understood yet. In general, the possible sensing mechanism for these materials can be related to the action between polarization-induced positive surface charges and ions in the electrolyte on the exposed region of GaN or AlGaN/GaN structures, and then, this will affect the surface charges of the devices. The change in the surface charge will result in the change in the carrier concentration of the channel causing a change in drain-source current. As a result, we can measure the pH of the solution by the related change in current. The expected advantages of using undoped-AlGaN/GaN as compared with doped structures are lower gate leakage current, lower pinch-off voltage and less noise due to the absence of any donor in the AlGaN. These are the reasons why many groups prefer non-modulation doped gallium nitride HEMT structures for electronic device applications [11,12].

This paper presents our investigation on pH-sensing characteristics of open-gated undoped AlGaN/GaN HEMT structures. We have investigated the basic transistor characteristics and liquid-phase sensing capability of open-gated devices with unpassivated undoped-AlGaN surfaces in aqueous solutions. The results obtained seem to open up the feasibility of cointegration with AlGaN/GaN HEMT circuits for sensor network applications.

## 2. Experimental

**Figure 1.**
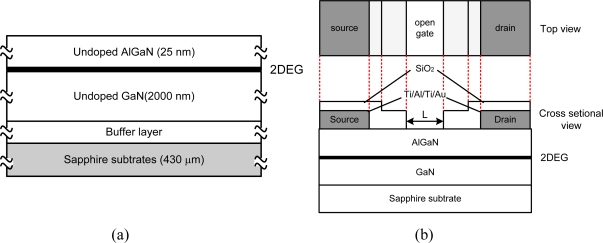
**(a)** Schematic of material layer structure (cross-sectional view) and **(b)** schematic of device structure (top and cross-sectional view).

Figure 1(a) shows the material structure. The AlGaN/GaN samples were grown by metal organic chemical vapor deposition (MOCVD) on 430 μm c-plane sapphire substrates. The thermal expansion coefficient of the sapphire substrate is close to those of aluminium oxide or aluminium nitride ceramics frequently used as packaging materials for high temperature sensors. Therefore, the complicated packaging technologies for high temperature sensors can be simplified.

As shown in Figure 1(a), the epitaxial structure consists of a 30-nm-thick GaN buffer layer, a 2-μm-thick undoped GaN layer and 25-nm-thick undoped-AlGaN barrier layer with an Al composition of 25%. The electron mobility and carrier sheet density of the two dimensional electron gas (2DEG) are 1,860 cm^2^/Vsec and 6.61 × 10^12^ cm^−2^, respectively, at room temperature. The GaN buffer is basically necessary to achieve a uniform Ga face polarity of the GaN epilayer across the entire substrate and also improves the structural quality of the following GaN-layer.

The schematic of the device structure is shown in Figure 1(b). The device fabrication process starts with 100-nm-thick SiO_2_ deposition using plasma-enhanced chemical vapour deposition (PECVD) at 280 °C with a SiH_4_/NH_3_/He gas system. This SiO_2_ dielectric layer plays a role as a mask for channel mesa formation in the following dry etching process. This dielectric mask layer is removed after that. A mesa etching is formed using inductive-coupled plasma (ICP)-assisted reactive ion beam etching with a Cl-based gas system consisting of BCl_3_, Cl_2_ and Ar. The etching pressure is 5 mTorr and the etching rate is around 0.1 μm/min. The drain and source electrodes are formed by deposition of Ti/Al/Ti/Au (20 nm/50 nm/20 nm/150 nm) multilayers, annealing process at 850 °C for 30 s under a flowing of N_2_ ambient by rapid thermal annealing system, and conventional lift off process. Although the present device is a two-terminal device, electrodes are called source and drain electrodes in this article so that the results on an open gated device, also known as a gateless device, can be correlated with the behavior of the gated device. The drain will be positively biased, and the voltage and current are called the drain-source voltage, *V_DS_*, and drain-source current, *I_DS_*, respectively.

**Figure 2.**
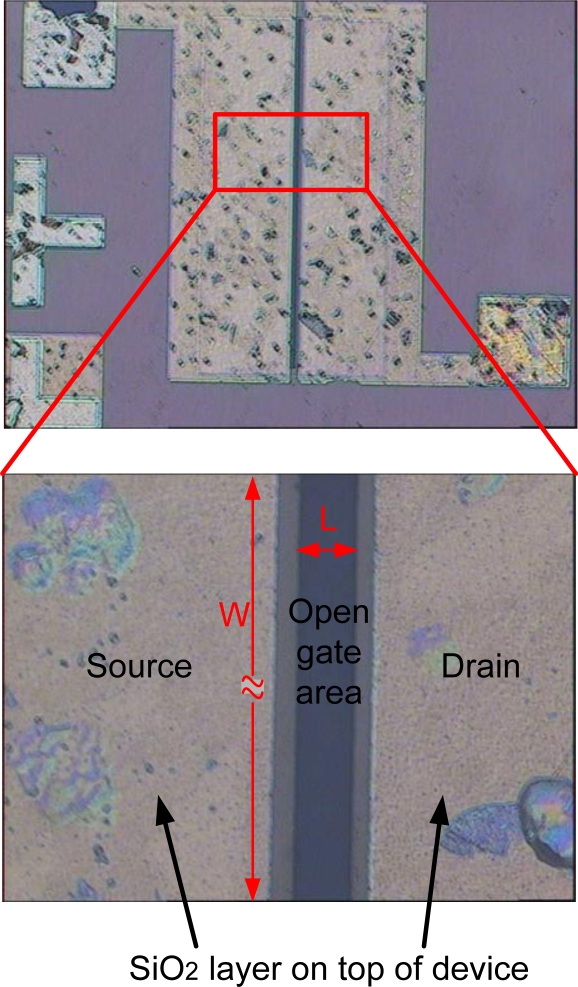
Photo of fabricated device (top view).

Next, the device surface is covered with 300-nm-thick SiO_2_ film using PECVD to prevent a chemical reaction between electrolyte and metal electrodes. Finally, the open-gate area, width, *W* of 490 μm and length, *L* of 40 μm, is defined through standard photolithography and wet etching processes in a buffered HF solution. The oxide layer is believed to be removed at this stage. However, a very thin oxide layer may be formed naturally after being exposed to the air. Since the experiment is done in the HCl-contained electrolyte, such a thin native oxide layer shall be etched out upon immersing in the electrolyte [13]. Therefore, the effect of native oxide layer on the sensing response is neglected in this study. The fabricated device is shown in Figure 2.

**Figure 3.**
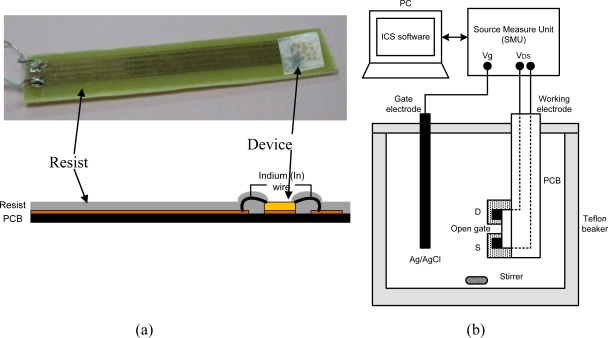
**(a)** Photo and schematic of sample holder and **(b)** schematic of the electrochemical system and measurement circuit.

Figure 3(a) shows a photo and schematic of sample holder. The sample is mounted using photoresist on a printed circuit board (PCB) which has a copper contact pad for the sample and copper conductor strips for source and drain connection. Wire bonding is made using Indium wire, as shown schematically in Figure 3(a). Photoresist is applied carefully on the wires and all metal contact areas, keeping only the open-gate region exposed for interaction with the electrolyte. Figure 3(b) shows a simple electrochemical system and a measurement circuit consisting of three source measure units (Keithley 236 SMU) and lab view control system. The gate bias is applied from a source measure unit to the electrolyte/AlGaN interface at the open-gate area via an Ag/AgCl electrode. For pH-sensing measurements, we prepared a mixed solution of HCl and NaOH in de-ionized (DI) water. The pH values in solutions are measured using a digital pH meter (Fisher Acumet AB15) after calibration with standard reference solution. All measurements in solutions are performed at room temperature (25 °C) under light conditions.

## 3. Results and Discussion

**Figure 4.**
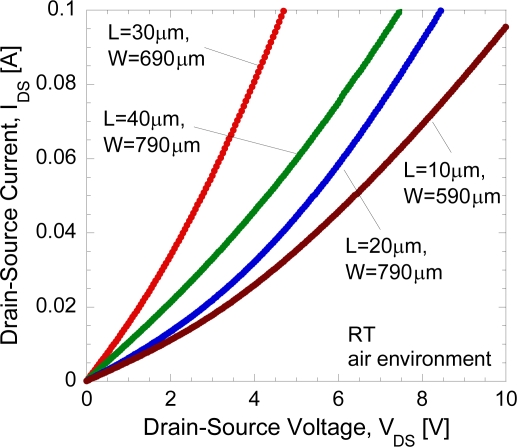
Typical *I_DS_−V_DS_* characteristics of the open gate HEMT in air condition.

The typical *DC* current-voltage (*I-V*) characteristics of the open-gated undoped-AlGaN/GaN HEMT structure in air-exposed environment under light condition at room temperature is shown in Figure 4.

It is clear that for all tested undoped-AlGaN/GaN samples, only the presence of the linear region of currents is observed, and no current saturation region is observed. It was reported in [14] by Hasegawa *et al*. that, in spite of an ungated structure, the curves of the tested Si-doped AlGaN/GaN samples show the presence of the linear and saturation regions of currents similar to those of the gated device. A possible mechanism of the appearance of current saturation and pinch-off behavior was proposed by Hasegawa *et al*. [14]. They deny the possibility of velocity saturation because the average electric field strength is too small to expect significant velocity saturation effect in such a long gate device. The proposed intepretation is that it is due to the presence of strong Fermi level pinning by surface states which tends to fix the surface potential at a particular position, and makes the entire surface behave like a virtual gate [14]. In fact, they have shown that the data could be reasonably well fitted to the theoretical *DC I_DS_−V_DS_* curves based on the gradual channel approximation. From our result, it can be simply said that the undoped AlGaN/GaN structure may produce very low Fermi level pinning by surface states. It also may due to large open-gate dimension which induces parasitic resistance. Thus, the drain current does not reach saturation, even up to 10 V.

**Figure 5.**
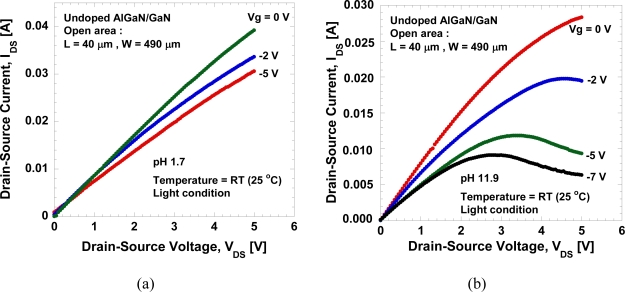
Typical *I_DS_−V_DS_* characteristics of the undoped open gated HEMT in **(a)** pH of 1.7 and **(b)** pH of 11.9.

Figure 5(a,b) show the typical *I_DS_−V_DS_* characteristics of the open-gate undoped AlGaN/GaN HEMT in a mixed solution of HCl and NaOH in DI water with pH values of 1.7 and 11.9, respectively. The measurement is done at room temperature in a room light environment. It can be seen in Figure 5(a) that the pinch-off behavior is hard to achieve in low pH solution compared to high pH solution. In addition, it is observed that large leakage current exists during measurement in low pH solution compared to high pH solution, and that data will be presented in the following figure. This large leakage current may be caused by the technical factors rather than the characteristics of the devices themselves. Despite of the existence of leakage current, the device shows conventional FET behavior with reasonably good gate controllability.

**Figure 6.**
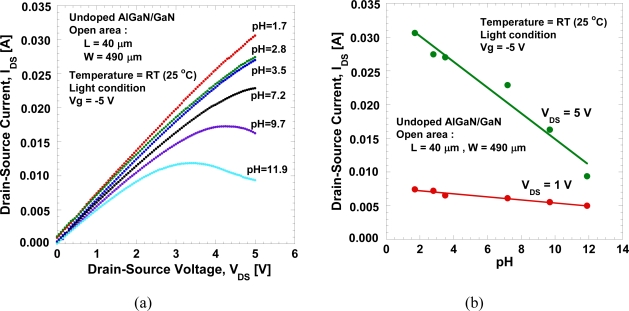
**(a)** *I_DS_−V_DS_* characteristics as a function of pH values and **(b)** measured *I_DS_* under *V_DS_* = 1 V and 5 V, and *V_g_* = −5 V

The *I_DS_−V_DS_* characteristics as a function of pH values is shown in Figure 6(a). The drain-source current decreases with the pH values, as expected. Figure 6(b) shows the drain-source current measured under *V_DS_* = 1 V and 5 V, and gate voltage, *V_g_* = −5 V. As expected, it clearly shows that the drain-source current decreases with the pH value. We obtained a large current change, ∼1.9 mA/pH or ∼3.88 mA/mm/pH at *V_DS_* = 5 V. In addition, a linear sensitivity is clearly observed, reflecting systematic change in potential at the AlGaN surface in the both linear and saturated bias regions. The measurements of the I-V characteristics are done repeatedly even after several days and are confirmed to produce similar characteristics.

Thus, it seems to show that undoped AlGaN/GaN open-gate HEMT devices are capable of distinguishing pH level and exhibit linear sensitivity. The exact mechanism of how these changes occur is still unknown, but a similar tendency has also been commonly observed in other reports [6]. It can be explained using electrolyte-insulator interfaces (SiO_2_, SiN*_x_*, Al_2_O_3_, AlN, *etc*.) in Si-based ion-sensitive FETs, where a site-binding model is generally accepted [15–17]. According to this model, hydroxyl groups (*M*OH: *M* represents Si or metals) are formed at insulator surfaces in contact with aqueous solutions, and can be dissociated to or combine with H^+^, depending on the H^+^ concentration and the equilibrium constants for the relevant reactions, as follows:

(1) $$M\text{OH}⇄MO^{-}+H^{+}$$

(2) $$M\text{OH}+H^{+}⇄M{\text{OH}}_{2}^{+}$$

When the H^+^ concentration decreases in solution, the right-direction reaction in the equilibrium Equation (1) becomes dominant, resulting in negative charges at the insulator surfaces due to deprotonized hydroxyls (*M*O^−^). On the other hand, the increase of H^+^ can induce positive charges at the surfaces due to protonized hydroxyls (*M*OH_2_^+^), represented by Equation (2). This leads to pH dependent net charge at the insulator surfaces, and the liquid-solid interfacial potential thereby follows the Nernst equation.

**Figure 7.**
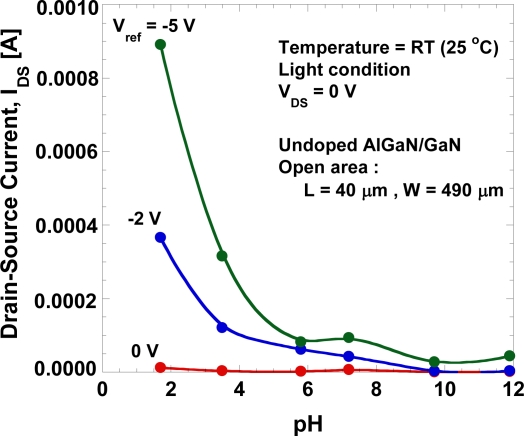
Drain-source current as a function of the gate voltage at *V_DS_* = 0 V.

**Figure 8.**
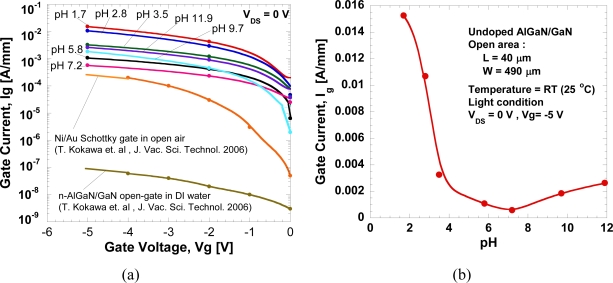
**(a)** Gate-leakage characteristics of the open gate undoped AlGaN/GaN HEMT at *V_DS_* = 0 V and **(b)** Changes of gate leakage current at various pH value.

Figure 7 shows the drain-source current at *V_DS_* = 0 V as a function of the gate voltage. A large drain-source current is present at low pH value and it increases with the negative gate voltage although no drain-source voltage is applied. Figure 8(a) shows the gate-leakage characteristics of the open-gate undoped AlGaN/GaN HEMT as a function of pH value. For comparison, the gate-leakage characteristics of the open-gate n-doped AlGaN/GaN HEMT in de-ionized water and a typical *I_GS_−V_GS_* curve of the Ni/Au Schottky-gate HEMT in air obtained from [6] are also shown together. The fabricated device shows a large leakage current and it increases with the decrease of pH value.

Figure 8(b) shows the magnitudes of gate-leakage current under various pH value at *V_DS_* = 0 V and *V_g_* = −5 V. As shown in Figure 8(b), gate current shows a drastic reduction from pH of 1.7 to 7.2, but increases from 7.2 to 12. This results show that the leakage-current depends strongly on the concentration of H^+^ ions in the electrolyte. We believe that this large leakage current may be caused by technical factors rather than the characteristics of the devices themselves. Because the sensor is wetted by the liquid electrolyte, it is critically important to isolate its electrical contacts for source and drain from the test liquid sample for reliable measurements. The adhesion of the photoresist is a major issue because with repeated use the photoresist may wear off, exposing the wires and pads, and causing device malfunctioning or gate-leakage. Measurement under light conditions may also enhance the density of carriers (electrons and holes) in the electrochemical system and contribute to the gate leakage.

Due to large leakage current where it increases with the negative gate voltage, the Nernstian-like sensitivity cannot be determined as normally reported by the other researchers [6]. Although there are imperfections in the device preparation, the fabricated devices work very well in distinguishing the pH levels, therefore, the fabricated open-gate undoped-AlGaN/GaN structure is expected to be suitable for pH sensing applications.

## 4. Conclusions

This investigation shows that undoped-AlGaN/GaN open-gate HEMT devices are capable of stable operation in aqueous electrolytes and exhibit linear sensitivity under light conditions. A high sensitivity of 1.9 mA/pH or 3.88 mA/mm/pH at *V_DS_* = 5 V is obtained. However, due to large leakage current where it increases with the negative reference gate voltage, the Nernstian-like sensitivity cannot be determined. Further improvements in device preparation and measurement under dark conditions is likely to improve the device performance in term of current leakage suppression. The fabricated open-gate undoped-AlGaN/GaN structure is expected to be suitable for pH sensing applications. In this paper, the response rate measurement which can be determined from transient time characteristics as normally reported was not carried out. This is due to the limitations of the present measurement set-up. The transient time characteristic measurements to determine the response speed will be carried out in the future after improving the sample preparation technique and measurement set-up. This work is underway.
